# Effects of cognitive-behavioral and psychodynamic-interpersonal treatments for eating disorders: a meta-analytic inquiry into the role of patient characteristics and change in eating disorder-specific and general psychopathology in remission

**DOI:** 10.1186/s40337-021-00430-8

**Published:** 2021-06-26

**Authors:** Leif Tore Moberg, Birgitte Solvang, Rannveig Grøm Sæle, Anna Dahl Myrvang

**Affiliations:** Department of Psychology, UiT The Arctic University of Norway, Huginbakken 32, N-9037 Tromsø, Norway

**Keywords:** Eating disorders, Cognitive-behavioral therapy, Psychodynamic therapy, Interpersonal therapy, Treatment effect, Psychopathology, Remission, Meta-analysis, Regression

## Abstract

**Background:**

Cognitive behavior therapy (CBT) and psychodynamic-interpersonal therapies (PIT) are two widely used and conceptually different outpatient treatments for eating disorders (EDs). To better understand how these treatments works, for whom, and under what circumstances, there is a need for knowledge about how outcomes are affected by diagnosis, comorbidity, changes in psychopathology, and study design.

**Method:**

Reports on the effects of CBT and PIT for eating disorders were searched. Rates of remission and changes in ED specific- and general psychopathology were computed. Regression models were made to predict event rates by changes in specific- and general psychopathology, as well as ED diagnosis and study design.

**Results:**

The remission rate of CBT for binge eating disorder was 50%, significantly higher than the effect for other diagnostic groups (anorexia = 33%, bulimia: 28%, mixed samples 30%). The number of studies found for PIT was limited. All effect sizes differed from zero (binge eating disorder = 27%, anorexia = 24%, bulimia = 18%, mixed samples = 15%), but the precision of the estimates was low, with some lower-bound confidence intervals close to zero. For CBT, change in ED specific psychopathology predicted remission only when controlling for ED diagnosis, while change in general psychopathology did not predict remission at all. The predictive value of change in psychopathology for PIT, and the potential impact of comorbid personality disorders could not be analyzed due to a lack of studies. There was no difference in effects between randomized controlled trials and observational studies.

**Conclusions:**

CBT showed consistent remission rates for all EDs but left a substantial number of patients not in remission. Extant evidence suggest that PIT is not consistently effective in achieving remission for patients with EDs, although this finding is uncertain due to a small number of eligible studies. A group of patients with eating disorders may, however, require therapy aimed at strengthening deficits in self functions not easily ameliorable by cognitive behavioral techniques alone. Further research should be aimed at identifying treatment interventions that helps patients change behavior, while strengthening self-functions to substitute eating-disordered behavior in the long-term.

**Supplementary Information:**

The online version contains supplementary material available at 10.1186/s40337-021-00430-8.

## Plain English summary

To help people with eating disorders (EDs) recover it is important to know what makes therapies effective or not. Therefore, we summarized the effects of two common therapies for eating disorders, cognitive behavior therapy (CBT) and psychodynamic-interpersonal therapy (PIT) and examined how ED diagnosis, comorbid personality disorder and changes in psychopathology could influence ED remission. We found that CBT was most consistently effective, with about 1/3 of patients in remission for anorexia, bulimia, and mixed samples, and 50% for patients with binge eating disorder. The effects of PIT were uncertain due to a lack of studies and could be only marginally effective. In CBT, changing the patients’ eating disordered thoughts or their depression or anxiety was not associated with ED remission. We discuss why this may be and suggest that CBT may be more effective because it manages to engage a subgroup of patients who are motivated to change and less depressed or anxious. Thus, patients with more severe symptoms may benefit less. We conclude that CBT may be necessary to help people recover from eating disorders, but that some patients may still require interventions aimed at strengthening self-functions to substitute eating disordered behavior in the long-term.

**Supplementary Information:**

The online version contains supplementary material available at 10.1186/s40337-021-00430-8.

## Background

The process of psychotherapy for eating disorders (EDs) are complicated by several features of these multifaceted psychiatric states. Rates of dropout from treatment range from 20 to 73%, and patients often have chronic courses of illness [1, 2]. EDs are associated with significant medical complications [3], and increased mortality [4]. There are different theoretical understandings of EDs, pertaining to their etiology and maintaining factors, and thus how best to address the difficulties patients face in treatment.

Although different diagnostic criteria are listed in the Diagnostic and Statistical Manual of Mental Disorders, (DSM-5) [5] for three discrete subtypes of EDs; anorexia nervosa (AN), bulimia nervosa (BN), and binge eating disorder (BED), diagnostic crossover between these diagnoses over time is a common phenomenon [6, 7]. Adding to the complexity of the clinical state of the patient with an ED are varying degrees of interpersonal difficulties and other comorbid psychiatric states, making several clinical features relevant to treatment and prognosis. Personality disorders (PDs) have been estimated to be comorbid with AN and BN in 50% of the cases [8]. Also, a large comorbidity survey for EDs [9] showed high rates of comorbid disorders (depressive, anxiety and substance use) across ED diagnoses (56% for AN, 94% for BN and 78% for BED). For patients with EDs, psychiatric comorbidities [10], and interpersonal difficulties, e. g. in the form of excessive social dominance, coldness, self-sacrifice or non-assertiveness [11] are associated with poor outcomes of psychotherapy, and persistence of eating-disordered symptoms.

Two historically prominent theoretical frameworks for understanding the psychopathology present in EDs are (a) the cognitive-behavioral model, adapted to the symptomatology and clinical presentations of EDs [12, 13] and (b) the psychodynamic [14] or interpersonal [15] models converging on the emphasis of the role of others in the development of the self, and relating psychopathology to a developmentally based deficit in self-functions. Regarding the treatment of EDs, these two traditions are distinguished by the extent to which they target eating-disordered cognitions and behaviors - what makes EDs special, or aspects of self-functions that are pertinent to all psychiatric disorders. The focus of this meta-analytic review was the treatment effects of these theoretically different treatment approaches.

According to the transdiagnostic cognitive-behavioral model of EDs [13], eating-disordered behaviors are aimed at controlling body weight and shape, and include dietary restriction, and compensatory strategies such as laxative use, vomiting and excessive exercise. For some these efforts results in occasional loss of control, characterized by subjective or objective episodes of binge-eating. All eating disordered behaviors are assumed to be driven by the core eating-disordered cognitions, i.e., the over-evaluation of shape and weight, and their control. For patients with EDs, self-evaluation is based largely on the extent to which they can control their shape and weight. The cognitive and behavioral traits are assumed to be mutually reinforcing and self-perpetuating maintaining mechanisms seen in AN, BN and BED [13]. Cognitive behavioral therapies aim to modify the cognitive triangle consisting of thoughts, emotions, and behavior by means of cognitive restructuring, and actively altering behavioral patterns [16, 17].

On the other hand, EDs can be regarded as disorders of the self. Psychodynamic and interpersonal theories have emphasized that deficiencies in self-cohesion, self-worth and self-agency challenge a person’s ability to contain emotional experiences and needs as real and legitimate, and understanding the affective, motivational, and cognitive states of the self and others [18–20]. Manifestations of these deficiencies may include emotion dysregulation, interpersonal difficulties [21] and eating disordered behavior [22, 23]. In people with EDs deficient self-functioning has been associated with general psychopathology such as anxious and depressive features [24, 25]. Eating disordered behaviors have been found to be effective strategies for regulating emotions [26] interpersonal relations and sense of self-worth and -agency [27, 28]. Experienced emotional distress may thus be an important maintaining factor, linking deficiency in the patient’s self-functions with the ED specific cognitive and behavioral features of EDs. The aim of psychodynamic-interpersonal therapy is to heighten the patient’s awareness, acceptance, and tolerance of affective experiences. Furthermore, the aim is to help the patient to integrate and contain previously disavowed affective and motivational content into the sense of self [14].

The National Collaborating Centre for Mental Health [29] recommends outpatient psychotherapy as a first-line treatment for EDs. Symptom-focused CBT is recommended for AN, BN and BED. For AN, it is also recommended using psychodynamic or interpersonal therapy approaches, but no specific therapy is recommended over another.

Treatment effects for CBT relative to other treatments have been established through several meta-analyses of randomized controlled trials (RCTs). CBT has demonstrated effectiveness in reducing eating-disordered cognitions [30] depressive symptoms [31] and increasing quality of life [32]. Furthermore, reduction of ED psychopathology predicted the reduction of behavioral symptoms for BN and BED samples [30], and reduction of binge/purge symptoms have been found to predict greater reduction of depressive symptoms in BN samples receiving CBT, compared to other treatments [31]. These findings lend preliminary support for the cognitive-behavioral model of EDs, and thus the core behavioral and cognitive symptoms as principal targets of therapeutic interventions.

Inferences as to the effect of other specified therapeutic approaches have, however, been difficult to make from meta-analyses of RCTs, e.g., [30–35] because effect sizes have been based on differences between treatment arms containing heterogenous interventions (e.g. different combinations of active experimental treatments, multimodal interventions and different variants of treatment as usual or active psychotherapy control conditions). Rates of ED remission, in terms of abstinence from ED behaviors have, however been synthesized in two meta analyses of RCTs for BN [36] and BED [37]. For BN, the rate was 30% and for BED 45%. Such figures are to date missing for AN. CBT was found to yield the highest ED remission rates for BN and interpersonal therapy for BED.

While RCTs are widely regarded as the gold standard for establishing the efficacy of a psychotherapy, concerns have been raised that some features of RCTs may compromise their external validity, e.g., restrictive patient inclusion criteria regarding comorbidity and other psychopathology, and strict adherence to a predefined therapy protocol [38]. Knowing the wide range of problems often associated with EDs, the standardization of both presenting problems and therapy protocol may prevent generalization of therapy outcomes to real-world clinics. Including observational studies from real-world clinical work allows for a comparison between the results of the different research approaches and can inform on how the efficacy of therapies established through RCTs translate to real-world clinical settings.

Knowledge is to date incomplete as to the effects of treatment alternatives to CBT, and the extent to which they work differently from CBT. Specifically, the existing literature on the treatment of EDs lacks meta-analytic reports and discussions on the effect of cognitive behavioral therapy and psychodynamic-interpersonal therapy in comparison, where 1) outpatient psychotherapy is the only intervention patients receive, and 2) each treatment modality is searched for specifically and thoroughly and 3) the impact of psychological changes on ED remission is assessed. Given the high rates of interpersonal problems and psychiatric comorbidity in ED presentations, and because anxious and depressive features may be linked to and maintain symptoms, it is important to understand the contribution of changes both in ED specific and general psychopathology to ED remission. Also, knowing the role of change in psychopathology following different psychotherapeutic approaches could help inform which aspect of psychopathology are most closely related to ED remission and how to target them most effectively.

The aim of this meta-analytic review is therefore to shed light on how these two specific and conceptually different treatment approaches work for patient samples with varying presentations of EDs in both RCTs and observational studies. To this end we raise three research questions:

1. What are the rates of ED-remission for CBT and PIT in outpatient settings, and do they differ between ED-diagnoses and people with/without PDs?
2. How does change in ED specific and general psychopathology affect ED remission?
3. How does study design (RCT vs. observational studies and follow-up time) affect the observed ED remission rates?

## Method

This meta-analytic review was reported in accordance with the Preferred Reporting Items for Systematic Reviews and Meta-Analyses guidelines [39], and was submitted for pre-registration in February 2020 at the International Prospective Register of Systematic Reviews [40]. All analyses were planned before the systematic searches, literature review and data extraction were performed. However, some pre-registered analyses could not be performed due to a lack of studies containing the relevant treatment arms, variables, and outcomes. Between-group effect sizes comparing CBT and PIT could not be synthesized as planned. Furthermore, mediation analyses with change scores, and interaction analyses with treatment approaches and patient characteristics could not be performed.

### Search strategy

Electronic databases that were searched were PsycInfo, Embase, Medline, Proquest Dissertations and Theses, and Cinahl. The searches were performed the 12.02.2020. Three search strings were constructed for the constructs “eating disorders”, “cognitive behavior therapy”, and “psychodynamic-interpersonal therapy”, respectively, each string consisting of several terms. The search strings representing the treatment approaches were first combined with the operator OR, then combined with the construct “eating disorder” with the operator AND. The complete search strategy is attached in Additional file 1: appendix B.

### Study selection and data management

Reports were pooled across databases and reviewed. Data from included studies were extracted by the first and second author independently. By the end of data extraction, results from the two authors were cross-checked. Discrepancies in results were solved by checking articles for the correct values. As EDs first was included as an independent chapter in DSM-3 in 1980, records older than 1980 were excluded.

### Primary study risk of bias assessment

Results of meta-analyses are susceptible to biases inherent in the design of the primary studies used. In the present meta-analysis, the studies included were rated using the quality assessment tool for quantitative studies (QATQS), developed by the Effective public health practice project [41]. This is a validated quality assessment tool used to rate the methodological quality of primary studies on 7 domains; selection bias, study design, confounders, blinding, data collection methods, withdrawals and drop-outs, intervention integrity, and data analyses, along with a global quality rating. The methodological strength of each domain is rated as strong, moderate, or weak, according to standardized criteria. Furthermore, measures were taken to reduce the impact of detection bias and attrition bias especially. To reduce inflation of effect sizes due to attrition bias, intention-to-treat analyses (ITT) were always used for ED remission, i.e., the number of patients in remission were compared to the number of patients randomized/admitted to treatment to begin with. Thus, patients who dropped out of treatment were always considered not in remission. Furthermore, primary studies are likely to contribute to detection bias if the definition of the desired outcomes of interventions are based on subjective ratings. Using objective criteria reduces this problem [42]. Therefore, definitions of remission based on non-blinded clinician ratings of clinically significant change, significant improvement or absence of an ED diagnosis were considered to contribute to detection bias.

### Eligibility criteria

During screening, all references to original papers on the treatment of EDs were considered for full text review, whether published or unpublished. To be considered eligible for final inclusion, the reports had to

- Provide information to calculate the event rate for the proportion of patients in ED remission
- Include a clinical trial of efficacy or observational study of treatment effectiveness
- Include at least one psychotherapeutic intervention that had a cognitive-behavioral focus or a psychodynamic-interpersonal focus
- Be directed to outpatients with a diagnosed ED.

Exclusion criteria in the full-text review were:

- Multimodal therapies combining, e.g., milieu therapy, medication, exercise; treatments combining aspects of CBT and PIT.
- Interventions not targeting the cognitive or psychodynamic-interpersonal aspects of EDs, e.g., exposure and response prevention, dietary advice or specialist supportive clinical management
- Treatments broader in scope than CBT or PIT, e.g., dialectical behavior therapy, and acceptance and commitment therapy.
- Unavailability of data to calculate effect sizes based on ITT, i.e., only data for completers were given.
- Ratings of ED remission was subjective, or used any other definition than abstinence for 28 days for BN and BED, and weight restoration to 85% IBW or a minimum BMI of 17.5 for AN.

### Data extraction and coding

#### Effect size calculation

All effect sizes were coded across two time-points: Pre-treatment (t0) and 12 months follow-up (t1). Because relapse rates are high for EDs, 12 months follow up was used to assess treatment effects that can be said to be stable over time. If outcome assessments were available for several time-points after the end of treatment, the time-point closest to 12 months was prioritized. If follow-up assessment was unavailable, end of treatment assessment was used and coded as 0.

#### Primary outcome variable

The number of patients intended to be treated at t0, and the number of patients in ED remission at t1 were extracted. ED remission was defined as the proportion of patients in the treated sample that has undergone weight normalization (AN-samples), cessation of compensatory behaviors (AN- and BN-samples), and cessation of bingeing at t1 (BN- and BED-samples). Patients unavailable for follow-up were considered not in ED remission.

#### Treatment approaches

Effect sizes were calculated for cognitive-behavioral therapy or psychodynamic-interpersonal therapy separately. The CBT approach was included and coded based on the focus on dysfunctional thoughts, beliefs and attitudes regarding eating, body shape and weight, and how these relate to behavior and emotions. The PIT approach was included and coded according to the definition by Blagys & Hilsenroth [43].

#### Predictors

##### Change in psychopathology

Furthermore, two secondary outcome variables were coded to be used as predictors for the main outcome variable, ED remission. Standardized within group changes in the form of Cohen’s *d,* were computed based on means and standard deviations at t0 and t1, or from correlations or *p*-values for pre-post changes at t0 and t1. Computing Cohen’s *d*, the correlation between the pre and post measures were set to .70, which is considered sufficiently close to the test-retest reliability of many psychometric scales [44].

For ED specific psychopathology, scales such as the Eating Disorder Examination (EDE) were preferred if primary studies reported several measures. This instrument consists of four subscales: restraint, eating concerns, shape concerns, and weight concerns, assumed to encompass the specific ED-psychopathology according to the cognitive-behavioral model of EDs. In studies where other instruments were used for measuring specific psychopathology, each subscale was evaluated in terms of relevance to ED specific psychopathology. Second, change in general psychopathology was quantified using assessment scales for depressive (e.g., BDI, HAM-D) or anxious (e.g., STAI-S, STAI-T, HAM-A) symptomatology. In cases where several subscales were reported, composite change scores were made from subscale scores measuring specific or general psychopathology.

##### Patient characteristics

First, ED diagnosis was coded as either AN, BN, BED, or mixed samples. Second, comorbidity was coded as the percentage of patients in the treated sample with a PD diagnosis.

##### Study design

To address the question whether results are comparable between RCTs and observational studies, study design was coded for each sample. The studies were coded as either RCTs or observational studies. Furthermore, as the follow-up time for ED remission varied in the studies, the time from end of treatment to follow-up were coded in the unit of months. Assessments at 12 months follow-up were always used if available. If not, the assessment time closest to 12 months was preferred.

### Data synthesis and meta-analysis

Meta analyses were performed by using the Comprehensive Meta-Analysis Software version 3 [45]. All meta-analytic models were constructed with effect sizes weighted by their inverse variance, assuming random effects, as is recommended when the true treatment effects reported by studies are expected to vary [46].

To answer question 1, within-group summary effect sizes were calculated for individual treatment arms where CBT or PIT were delivered, using event rates for the proportion of patients in ED remission. Because effect sizes were derived from studies with different designs and patient samples, significant statistical heterogeneity was expected and subjected to examination. Specifically, effect sizes were calculated for each of the ED diagnoses and differences between diagnoses statistically assessed.

To answer question 2, Cohen’s *d* for pre-post changes in psychopathology were computed and the impact of change in ED specific- and general psychopathology on ED remission was assessed for each of the ED diagnoses. The values of change scores were centered as is recommended for continuous variables used in multiple regression with categorical variables [47]. Regression models were made for each treatment approach, where ED remission rates were independently predicted by change scores. Furthermore, the relative importance of each hypothesized mediator was examined by comparing their respective regression coefficients, the variance explained by, and significance of the addition of this variable to the model. Also, ED diagnosis was assessed as a potential moderator of these effects.

To answer question 3, regression analyses of ED remission on study design (RCTs vs observational) and follow-up time were performed, and regression coefficients and variance explained statistic assessed.

### Publication bias assessment

One vulnerability of meta-analyses is the potential presence of publication bias, i.e., if studies with weak or non-significant effects are not published and therefore not included in the analysis. Publication bias has been identified as a problem in both psychological and medical research [48] but is unreliable to test with one method only. The use of several methods is therefore recommended [49]. To test for publication bias, we used funnel plots to visually assess the presence of publication bias and Egger’s regression for examining correlations between sample size and estimated effect sizes [50]. Using the Duvall and Tweedie’s Trim and fill method [51] for imputing missing studies, the adjusted effect sizes for CBT and PIT were examined for all outcomes.

## Results

### Study characteristics

**Fig. 1:**
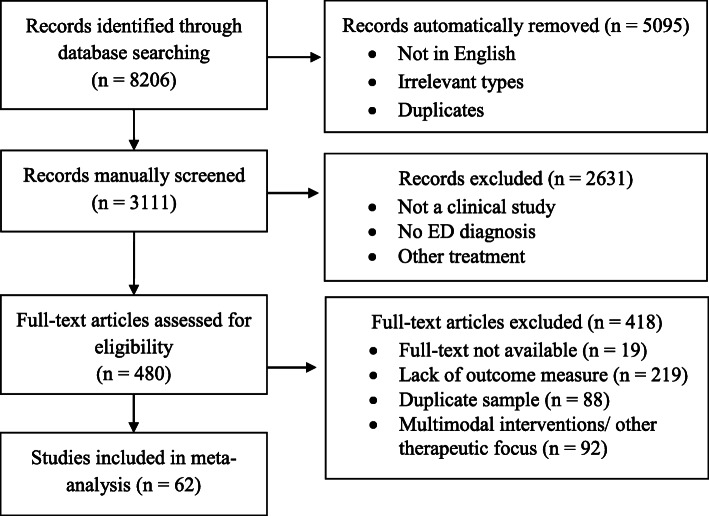
PRISMA flow diagram for the systematic literature review

Figure 1 displays the results from the systematic literature searches and the following review process. Table E1-E4 in Additional file 1: Appendix E contains complete descriptions of characteristics for all included studies, and a complete reference list of included studies is attached in Additional file 1: appendix A. After removal of irrelevant reference types (e.g., qualitative studies, books, reviews, comments, editorials, and papers in other languages than English), 3111 references were screened for eligibility.

### Risk of bias assessment

Definitions of ED remission varied widely across studies, even within diagnostic categories. Two hundred nineteen studies were excluded because outcome measures were not relevant or did not pass the risk of bias assessment. Of these, 60 met all inclusion criteria and had relevant outcomes but were excluded because they were deemed as having high risk of detection bias. Most of these studies used other criteria relating to time of abstinence, did not state the length of abstinence, defined ED remission as absence of the presenting diagnosis, or used other qualitative categories such as “good outcome” or “significantly improved”. After full text review, 62 studies, with 76 samples met the inclusion criteria. Table 1 displays characteristics of the sample of studies included in analyses.

**Table 1:** Characteristics for the included samples and number of samples across variables (*K* = 76)

Variable	Value	k (samples)
Treatment approach	CBT	66
	PIT	10
ED diagnosis	AN	20
	BN	19
	BED	17
	Mixed	20
Proportion with PD	Percentage	10
Design	RCT	37
	Observational	39
Follow-up time	Continuous	71

Note. *k* number of samples, *CBT* Cognitive behavior therapy, *PIT* Psychodynamic-interpersonal therapy, *AN* Anorexia nervosa, *BN* Bulimia nervosa, *BED* Binge eating disorder, *Mixed* Mixed diagnoses, *RCT* Randomized controlled trial, *Observational* Observational study

Of the included studies, 23 (37%) were rated with strong, 31 (50%) with moderate, and 8 (13%) with weak global methodological quality on the QATQS. In all included studies it was possible to calculate intent-to-treat remission rates; the therapies were sufficiently described and carried out and did not include any confounding interventions. The data collection, analyses, and integrity of intervention domains were therefore all rated as strong. Selection bias was the domain in which the most studies were rated as methodologically weak (*k* = 25, 40%)*.* An equal number was rated with moderate quality, and 12 (20%) were rated as strong. For the design quality domain, 25 studies (40%) were rated with strong methodology, i.e., they were RCTs where the methods of randomization were clearly stated, 35 studies (57%) were rated as moderate (described as RCTs, but with an unclear method of randomization, or observational studies), 2 (3%) were rated as weak, i.e., allocation to treatment was not described. On the confounders domain 19 studies (30%) was rated as strong, i.e., RCTs which reported no statistical differences between groups at baseline, 37 studies (60%) as moderate, observational studies with no obvious confounders, and 6 studies (10%) as weak. On the drop-out domain, 12 studies (19%) were rated with strong, 39 (63%) with moderate, and 11 (18%) with weak methodological quality, i.e., with 40% or more of participants dropping out of treatment. See Tables F1-F4 in Additional file 1: Appendix F for the quality rating on each domain for each individual study.

### Treatment effects for CBT and PIT by ED diagnosis and comorbidity

The event rates for ED remission for CBT and PIT are presented in Table 2 for each of the eating disorders individually. For CBT, ED diagnosis was a significant independent predictor of logit event rate for remission (*Q*(3) = 25.53, *p* < .001), explaining 29% of total variance. 50% of the patients with BED achieved remission, which is the highest remission rate among the ED diagnoses. Thus, CBT is most effective in treating BED compared to AN, BN and mixed diagnoses samples. For PIT, ED diagnosis did not significantly predict logit event rate for remission (*Q*(3) = 1.26, *p* = .739), and did not explain any of the variance. There were not enough studies (k = 10) distributed across treatments and diagnoses to evaluate how the proportion of patients with a comorbid PD in the sample affects remission rates.

**Table 2:** Event rates for ED remission by treatment approach and diagnosis (K = 76)

			95% CI		
	*k*	Event rate	Lower	Upper	*I*^2^
CBT					
AN	17	.33	.276	.395	77%
BN	15	.28	.224	.339	29%
BED	15	.50	.424	.568	0%
Mixed	19	.30	.251	.352	83%
PIT					
AN	3	.24	.120	.431	52%
BN	4	.18	.092	.321	28%
BED	2	.27	.129	.492	0%
Mixed	1	.15	.043	.416	0%

*Note*. *k* = number of samples, *I*^2^ = the percentage of between-study heterogeneity not due to sampling error; *AN* Anorexia nervosa, *BN* Bulimia nervosa, *BED* Binge eating disorder, *Mixed* samples consisting of more than one ED diagnose, *CBT* Cognitive behavior therapy, *PIT* Psychodynamic-interpersonal therapy

### Meta-regression for remission on change in psychopathology

Table 3 displays results of meta regression of logit event rates for remission on change in specific and general psychopathology. For CBT samples, remission rates were not significantly predicted by change in specific psychopathology (*Q*(1) = 3.71, *p* = .054), leaving significant unexplained variance (*Q*(34) = 133.06, *p* < .001). The amount of total variance explained by the model was 4%. Remission rates were not significantly predicted by change in general psychopathology (*Q*(1) = .00, *p* = .946), leaving significant unexplained variance (*Q*(29) = 114.43, *p < .001*). The amount of total variance explained by the model was 0%. Neither specific nor general change predicted remission for either of the diagnostic subgroups.

**Table 3:** Meta regression of logit event rates for ED remission on change in psychopathology

	General change						ED specific change					
			95% CI						95% CI			
	*k*	*B*	Lower	Upper	*R*^2^	*p*	*k*	*B*	Lower	Upper	*R*^2^	*p*
CBT	31	−.03	−.870	.812	.00	.946	36	.42	−.073	.844	.04	.054
AN	4	−.54	−2.678	1.605	.00	.623	7	1.36	−.093	2.814	.37	.067
BN	9	.97	−.074	2.015	.46	.069	8	.40	−.444	1.252	.00	.350
BED	8	.03	−1.141	1.192	.00	.965	9	.11	−.527	.750	.00	.733
Mixed	10	.73	−.624	2.082	.13	.291	12	.41	−.053	.863	.13	.083

*Note*. *k* = number of samples; *B* = unstandardized regression coefficients; *R*^2^ = variance explained; *p* = significance level; *AN* Anorexia nervosa, *BN* Bulimia nervosa, *BED* Binge eating disorder, *Mixed* Samples consisting of more than one ED diagnose, *CBT* Cognitive behavior therapy

Because of nonsignificant coefficients for change in psychopathology and significant unexplained variance, the simple regression models were followed up with hierarchical regressions, testing ED-diagnosis as a potential moderator of the effect. Adding diagnostic subgroups to the model, ED specific change significantly predicted ED remission (*B* = .40, *p* < .001), and model fit was significantly increased (Q(3) = 42.05, p < .001). The entire model (*Q*(4) = 48.54, *p* < .001), explained 70% of the variance in ED remission.

A second model was also constructed for general change. Adding diagnostic subgroups to the model, general change did not significantly predict ED remission (*B* = .61, *p* = .073). However, model fit significantly increased (*Q*(3) = 31.21, *p* < .001), explaining 63% of the variance in ED remission.

For PIT samples, regression analyses of ED remission on ED specific and general psychopathology could not be performed due to an insufficient number of studies.

### Meta-regression for ED remission on design characteristics

Allocation to study (RCTs vs. non-RCTs) did not significantly predict logit event rate for ED remission (*Q*(1) = .47, *b* = −.102, *p* = .495), explaining 0% of the variance. Follow-up-time did not significantly predict logit event rate for remission (*Q*(1) = 1.88, *b* = .010, *p* = .171), explaining 6% of the variance.

### Publication bias assessment

Inspection of funnel plots (Additional file 1: Appendix C, Fig. C1) and Egger’s regression indicated that remission rates were similar in high-precision studies and low-precision studies, not indicative of publication bias. The Duvall and Tweedies trim and fill method suggested some adjustments in logit event rates for each of the treatment approaches but adjusting for publication bias did not significantly change the remission rates. For a report on publication bias assessment see Additional file 1: Appendix D.

## Discussion

This meta-analysis examined three research questions: First, we investigated the remission rates in outpatient CBT and PIT treatment for each of four ED diagnoses (AN, BN, BED, and mixed). We also wanted to investigate whether the results would differ between patients with/without PDs, but because of too few studies, this part of the question remains unanswered. Second, the predictive value of change in psychopathology for remission were examined; and third, the role of study design and follow-up time on the observed effects.

The results can be summarized as follows: For CBT, 66 samples were found, covering all the four different ED diagnoses. Remission rates for AN, BN and Mixed samples ranged from 28 to 33% with no significant difference between them. However, BED samples achieved a significantly higher ED remission rate of 50% with CBT. For PIT, a total of 10 samples were found. Remission rates ranged from 15 to 27% for the ED diagnoses, with no significant difference between them. Effects for the proportion of patients with PDs could not be estimated, due to lack of studies (k = 10) and an uneven distribution across treatment approaches and ED diagnoses. Reduction in ED specific- or general psychopathology did not independently predict higher rates of ED remission for CBT, all diagnoses taken together. However, when controlling for differences between diagnoses, change in ED specific-, but not general psychopathology emerged as a significant predictor of remission. Due to an insufficient number of studies, regression analyses of change in psychopathology could not be performed for PIT. Study design did not predict effect sizes for this sample of studies, nor did follow-up time.

### Treatment effects of CBT and PIT for remission by ED diagnosis

The remission rate of CBT for BED is 50% with confidence intervals (CIs) indicating effects of 42–57% which is significantly higher than the effects for AN, BN and Mixed samples. Confidence intervals ranged from 22 to 28% and up to 34–40 for BN and AN, respectively. There was no significant difference between these diagnoses.

Regarding the effects of PIT, all estimates are significantly different from zero, but the precision of the estimates is low for all diagnoses. The true remission rates for PIT, indicated by lower-bound confidence intervals, may be as low as 4–12% (for mixed samples and BED, respectively). The upper-bound CIs are 32–49% for (BN and BED respectively) with no significant difference between diagnoses.

Although no direct comparisons have been made between CBT and PIT in this study, the effects for the treatment approaches suggest that CBT were more consistently effective than PIT across all ED diagnoses. However, eating-disordered behavior persisted in many patients even after receiving this more consistently effective treatment.

The treatment effects for CBT described in this study are in line with treatment effects identified by Linardon et al. for CBT in BN [36] and BED [37] when using the same criteria for ED remission (i.e., 28 days abstinence from bingeing and purging). The present study is, however, the first meta-analysis of the proportion of patients with AN achieving weight restoration in outpatient samples receiving pure psychotherapeutic treatment and analyzing differences in treatment effects between diagnoses.

The finding that CBT was significantly more effective for BED than the other diagnoses, may indicate that the objective episodes of binge eating characterizing BED are more easily modifiable by cognitive-behavioral techniques than are the dietary restriction and purging behavior seen in AN, BN and many of the OSFED/EDNOS presentations in the mixed samples group.

### The role of change in psychopathology for ED remission in CBT

For CBT, change in ED specific psychopathology emerged as a significant predictor of ED remission only when controlling for differences between diagnoses. Such a finding contrasts with what may be expected by transdiagnostic symptom-focused CBT, possibly implying that the maintaining mechanisms and thus relevant targets for treatment interventions may not be uniform across all ED presentations. For instance, although not significant, change in ED specific psychopathology tended to be a more important predictor of change in AN compared to the other diagnostic subgroups, explaining 37% of the variance in ED remission.

Change in general psychopathology, i.e., anxious, and depressive features did not seem to be related to ED remission in CBT across ED diagnoses. Effects of change in general psychopathology was not significant, even when controlling for differences between ED diagnoses. For BN, however, change in general psychopathology tended to be more important than in the other diagnostic subgroups, explaining 47% of the variance.

These findings may indicate that the more consistent effects of CBT on the behavioral symptoms of EDs found in the present study may be conveyed through other mechanisms in the therapeutic process or be contingent on some patient factors. Among patient factors, higher motivation for change, fewer depressive features, fewer comorbidities and better interpersonal functioning have previously been found to predict better treatment outcomes [52]. Furthermore, the cognitive flexibility needed to produce cognitive and behavioral change may be weakened due to chronic malnourishment in some patients with AN, and depressive features in BN [53]. Thus, the effect of change in ED specific psychopathology on behavior may be contingent on neuropsychological health as well as differences in psychological functioning relating to the sense of self, e.g., motivation for change and levels of depression.

Some patients may experience significant ambivalence towards the prospect of change due to the ego-syntonic nature of the eating-disordered symptoms [54, 55] In patients where the sense of self is pervasively impaired (i.e., where there is significant lack of self-cohesion, and doubt in self-worth and self-efficacy), or the self is wholly dependent upon the over-evaluation of shape and weight [19], the working alliance to change behavioral aspects of the disorders in therapy may be lacking [56]. Because early change in eating-disordered behavior and cognitions [57, 58] is an important predictor of a favorable outcome of CBT, this could imply that some patients would at baseline be less likely to benefit from treatment than others.

### Strengths and limitations of the present study

Only studies reporting strictly operationalized variables regarding diagnosis, treatment approach, psychopathology and ED remission were included in this meta-analysis. Furthermore, only outpatient treatments based exclusively on cognitive-behavioral- or psychodynamic-interpersonal theory of psychopathology and therapeutic change were included. Reducing the confounding effects of multimodal interventions, this allows some insight into the purely psychological process of therapeutic change in EDs. To increase the ecological validity of the results, data from observational studies and grey literature were also included. Measures were taken to circumvent the effect of attrition bias for ED remission, as effect sizes were always based on intention-to-treat samples. Furthermore, the impact of detection bias was reduced by consistently using objective criteria for ED remission. The questions sought to be answered in this article were theoretically motivated and determined before starting data-collection. Such research practice is encouraged because it reduces the amount of reporting bias in the scientific literature [59].

Several features of this study may warrant caution in interpreting the results. The summary effect sizes reported in this study, i.e., event rates, are not controlled effect sizes and indicate only the proportion of patients in remission at follow-up after therapy. As such, a causal relationship between the therapeutic interventions and remission from EDs cannot be established, as several other factors extraneous to therapy itself may affect this outcome.

Furthermore, our sample of studies are characterized by a disproportionate distribution of CBT and PIT samples; CBT being six times more frequent than PIT. Exclusion of papers due to high risk of detection bias also disproportionally affected PIT studies, since these were few to begin with and a high proportion of them excluded. This limits the generalizability of the present findings regarding the effects of PIT. As the number of PIT studies were small for each of the ED diagnoses, it is not at the present possible to conclude with any certainty about its effects.

Although the overall methodological quality of the studies included were satisfactory, there was risk of selection bias in a high proportion of the studies. This was due to screening processes excluding a high proportion of potentially eligible participants. Exclusive screening processes warrants concern about whether the selected samples truly represent the population in question, i.e., people with EDs.

Publication bias is a prevalent phenomenon in psychological research [60]. There was no evidence of publication bias in the effect sizes for remission. However, methods for detecting publication bias do not perform well when heterogeneity in the effect sizes is large as in this meta-analysis [48], and the results must therefore be interpreted with caution.

Baseline differences in patients psychological functioning, e.g., the severity of eating disordered psychopathology, depression, anxiety, and impairment in the sense of self is not accounted for in this study. We attempted to examine the role of comorbid PDs in remission. However, not enough studies which included or screened for this in patients were found to analyze the effect of treatment for patients with- and without PDs.

### Directions for future research

In this study we examined the effect of change in ED specific- and general psychopathology on remission and did not find a statistically significant effect for CBT. We suggested that some baseline patient characteristics (general- and ED specific psychopathology and interpersonal difficulties) may be responsible for this, i.e., that patients who are high in these traits may be less likely to benefit from treatment than those who are lower in these traits to begin with. This has, however, not been examined in the present study. Future meta-analyses could help inform on this question by including baseline traits as predictors of therapy outcomes.

To make PIT a legitimate treatment alternative to CBT there is a need for more RCTs and observational studies examining the efficacy/effectiveness of these treatments on objective outcome criteria. Also, more consistent definitions of ED remission and agreement on relevant outcome measures, across different treatment approaches would contribute to making comparisons between treatments possible. Studies on ED remission in PIT tend to use remission definitions pointing to those aspects of psychological functioning underlying eating-disordered behavior instead of behavioral definitions, as used by CBT studies. Perhaps, primary studies should use both definitions independently of treatment approach, making the outcomes possible to compare directly to each other.

Furthermore, only ten studies included or screened for PDs in the patient samples. Given that PDs are highly comorbid with EDs, it would be useful to know whether therapy is as effective for them or whether they may require other interventions instead/in addition to cognitive behavioral techniques or whether psychodynamic interventions have some benefit especially for these patients. Therefore, it would be informative if primary clinical studies included patients with PDs and reported the proportion of the sample they made up.

## Conclusion

Our findings suggest that CBT showed more consistent treatment effects than PIT, indicating that a therapeutic focus on the ED specific behavior, which is characteristic for CBT may be necessary to reliably produce behavioral ED remission. The results of the present study confirm, however, that EDs are multifaceted psychiatric states with need for a thorough understanding about precipitating and maintaining factors. Some of the findings in this study suggest that other aspects of psychological functioning than those pertaining exclusively to ED specific cognitions and behavior may affect treatment outcomes and warrant therapeutic attention; a) the effect of change in ED psychopathology was not sufficient to explain rates of ED remission, b) higher remission rates for CBT in BED than in the other ED presentations. Although the results of the present study suggest that CBT has the best effect on remission, there is a need to further investigate why change in ED-specific psychopathology and general psychopathology is not more strongly related to remission.

## Supplementary Information

**Additional file 1: Appendix A.**. References for primary studies included in the meta-analyses. **Appendix B.** Search strategy. **Appendix C.** Funnel plot. **Appendix D.** Publication bias assessment. **Appendix E.** Primary study characteristics sorted by diagnostic subgroup. **Appendix F.** Primary study quality assessment.

## Data Availability

The datasets generated and analyzed during the current study are available in the Open Science Framework repository [61], at https://osf.io/8mynp/

## Abbreviations

AN: Anorexia nervosa
BED: Binge eating disorder
BN: Bulimia nervosa
CBT: Cognitive behavior therapy
ED: Eating disorder
PD: Personality disorder
PIT: Psychodynamic-interpersonal therapy
RCT: Randomized controlled trial
