# Supplementary material for: Effects of cognitive-behavioral and psychodynamic-interpersonal treatments for eating disorders: a meta-analytic inquiry into the role of patient characteristics and change in eating disorder-specific and general psychopathology in remission
Source: J Eat Disord. 2021 Jun 26;9:74. doi: 10.1186/s40337-021-00430-8 (PMC8235811; doi:10.1186/s40337-021-00430-8)
Supplement: Supplementary file 1 — Additional file 1: Appendix A.. References for primary studies included in the meta-analyses. Appendix B. Search strategy. Appendix C. Funnel plot. Appendix D. Publication bias assessment. Appendix E. Primary study characteristics sorted by diagnostic subgroup. Appendix F. Primary study quality assessment. [file 40337_2021_430_MOESM1_ESM.docx]

**Appendix A: References for primary studies included in the meta-analyses.**

1. Agras WS, Crow SJ, Halmi KA, Mitchell JE, Wilson GT, Kraemer HC. Outcome predictors for the cognitive behavior treatment of bulimia nervosa: data from a multisite study. Am J Psychiatry. 2000;157(8):1302-8.

2. Agras WS, Walsh T, Fairburn CG, Wilson GT, Kraemer HC. A multicenter comparison of cognitive-behavioral therapy and interpersonal psychotherapy for bulimia nervosa. Arch Gen Psychiatry. 2000;57(5):459-66.

3. Aguera Z, Krug I, Sanchez I, Granero R, Penelo E, Penas-Lledo E, et al. Personality changes in bulimia nervosa after a cognitive behaviour therapy. Eur Eat Disord Rev. 2012;20(5):379-85.

4. Aguera Z, Riesco N, Jimenez-Murcia S, Islam MA, Granero R, Vicente E, et al. Cognitive behaviour therapy response and dropout rate across purging and nonpurging bulimia nervosa and binge eating disorder: DSM-5 implications. BMC Psychiatry. 2013;13:285.

5. Allen KL, Fursland A, Raykos B, Steele A, Watson H, Byrne SM. Motivation-focused treatment for eating disorders: a sequential trial of enhanced cognitive behaviour therapy with and without preceding motivation-focused therapy. Eur Eat Disord Rev. 2012;20(3):232-9.

6. Arcelus J, Whight D, Langham C, Baggott J, McGrain L, Meadows L, et al. A case series evaluation of a modified version of interpersonal psychotherapy (IPT) for the treatment of bulimic eating disorders: a pilot study. Eur Eat Disord Rev. 2009;17(4):260-8.

7. Bailer U, de Zwaan M, Leisch F, Strnad A, Lennkh-Wolfsberg C, El-Giamal N, et al. Guided self-help versus cognitive-behavioral group therapy in the treatment of bulimia nervosa. Int J Eat Disord. 2004;35(4):522-37.

8. Ball J, Mitchell P. A randomized controlled study of cognitive behavior therapy and behavioral family therapy for anorexia nervosa patients. Eat Disord. 2004;12(4):303-14.

9. Bandini S, Antonelli G, Moretti P, Pampanelli S, Quartesan R, Perriello G. Factors affecting dropout in outpatient eating disorder treatment. Eat Weight Disord. 2006;11(4):179-84.

10. Byrne SM, Fursland A, Allen KL, Watson H. The effectiveness of enhanced cognitive behavioural therapy for eating disorders: an open trial. Behav Res Ther. 2011;49(4):219-26.

11. Castellini G, Vignozzi L, Fisher A, Lelli L, Godini L, Maggi M, et al. Body Image Disturbance, Sexual Functioning and Hormonal Levels: A Longitudinal Study in Anorexia Nervosa Patients. J Sex Med. 2015;12:196-.

12. Chen E, Touyz SW, Beumont PJ, Fairburn CG, Griffiths R, Butow P, et al. Comparison of group and individual cognitive-behavioral therapy for patients with bulimia nervosa. Int J Eat Disord. 2003;33(3):241-54; discussion 55-6.

13. Cooper PJ, Steere J. A comparison of two psychological treatments for bulimia nervosa: implications for models of maintenance. Behav Res Ther. 1995;33(8):875-85.

14. Dalle Grave R, Calugi S, Doll HA, Fairburn CG. Enhanced cognitive behaviour therapy for adolescents with anorexia nervosa: an alternative to family therapy? Behav Res Ther. 2013;51(1):R9-R12.

15. Dalle Grave R, Calugi S, Sartirana M, Fairburn CG. Transdiagnostic cognitive behaviour therapy for adolescents with an eating disorder who are not underweight. Behav Res Ther. 2015;73:79-82.

16. Dalle Grave R, Sartirana M, Calugi S. Enhanced cognitive behavioral therapy for adolescents with anorexia nervosa: Outcomes and predictors of change in a real-world setting. Int J Eat Disord. 2019;52(9):1042-6.

17. Dare C, Eisler I, Russell G, Treasure J, Dodge L. Psychological therapies for adults with anorexia nervosa: randomised controlled trial of out-patient treatments. Br J Psychiatry. 2001;178:216-21.

18. Dingemans AE, Spinhoven P, van Furth EF. Predictors and mediators of treatment outcome in patients with binge eating disorder. Behav Res Ther. 2007;45(11):2551-62.

19. Fairburn CG, Cooper Z, Doll HA, O'Connor ME, Palmer RL, Dalle Grave R. Enhanced cognitive behaviour therapy for adults with anorexia nervosa: a UK-Italy study. Behav Res Ther. 2013;51(1):R2-8.

20. Fischer S, Meyer AH, Dremmel D, Schlup B, Munsch S. Short-term cognitive-behavioral therapy for binge eating disorder: long-term efficacy and predictors of long-term treatment success. Behav Res Ther. 2014;58:36-42.

21. Frostad S, Danielsen YS, Rekkedal GA, Jevne C, Dalle Grave R, Ro O, et al. Implementation of enhanced cognitive behaviour therapy (CBT-E) for adults with anorexia nervosa in an outpatient eating-disorder unit at a public hospital. Journal of Eating Disorders. 2018;6.

22. Garner DM, Rockert W, Davis R, Garner MV, Olmsted MP, Eagle M. Comparison of cognitive-behavioral and supportive-expressive therapy for bulimia nervosa. Am J Psychiatry. 1993;150(1):37-46.

23. Goldbloom DS, Olmsted M, Davis R, Clewes J, Heinmaa M, Rockert W, et al. A randomized controlled trial of fluoxetine and cognitive behavioral therapy for bulimia nervosa: short-term outcome. Behav Res Ther. 1997;35(9):803-11.

24. Gowers S, Norton K, Halek C, Crisp AH. Outcome of outpatient psychotherapy in a random allocation treatment study of anorexia nervosa. Int J Eat Disord. 1994;15(2):165-77.

25. Grilo CM, Masheb RM, Wilson GT, Gueorguieva R, White MA. Cognitive-behavioral therapy, behavioral weight loss, and sequential treatment for obese patients with binge-eating disorder: a randomized controlled trial. J Consult Clin Psychol. 2011;79(5):675-85.

26. Hay P, Touyz S, Arcelus J, Pike K, Attia E, Crosby RD, et al. A randomized controlled trial of the compuLsive Exercise Activity TheraPy (LEAP): A new approach to compulsive exercise in anorexia nervosa. Int J Eat Disord. 2018;51(8):999-1004.

27. Hilbert A, Petroff D, Neuhaus P, Schmidt R. Cognitive-Behavioral Therapy for Adolescents with an Age-Adapted Diagnosis of Binge-Eating Disorder: A Randomized Clinical Trial. Psychother Psychosom. 2020;89(1):51-3.

28. Hilbert A, Tuschen-Caffier B. Body image interventions in cognitive-behavioural therapy of binge-eating disorder: a component analysis. Behav Res Ther. 2004;42(11):1325-39.

29. Jenkins PE, Morgan C, Houlihan C. Outpatient CBT for Underweight Patients with Eating Disorders: Effectiveness Within a National Health Service (NHS) Eating Disorders Service. Behav Cogn Psychother. 2019;47(2):217-29.

30. La Mela C, Maglietta M, Lucarelli S, Mori S, Sassaroli S. Pretreatment outcome indicators in an eating disorder outpatient group: the effects of self-esteem, personality disorders and dissociation. Compr Psychiatry. 2013;54(7):933-42.

31. Le Grange D, Lock J, Agras WS, Bryson SW, Jo B. Randomized Clinical Trial of Family-Based Treatment and Cognitive-Behavioral Therapy for Adolescent Bulimia Nervosa. J Am Acad Child Adolesc Psychiatry. 2015;54(11):886-94 e2.

32. Lelli L, Castellini G, Cassioli E, Monteleone AM, Ricca V. Cortisol levels before and after cognitive behavioural therapy in patients with eating disorders reporting childhood abuse: A follow-up study. Psychiatry Res. 2019;275:269-75.

33. Lo Sauro C, Castellini G, Lelli L, Faravelli C, Ricca V. Psychopathological and clinical features of remitted anorexia nervosa patients: a six-year follow-up study. Eur Eat Disord Rev. 2013;21(1):78-83.

34. Lock J, Agras WS, Fitzpatrick KK, Bryson SW, Jo B, Tchanturia K. Is outpatient cognitive remediation therapy feasible to use in randomized clinical trials for anorexia nervosa? Int J Eat Disord. 2013;46(6):567-75.

35. McIntosh VVW, Jordan J, Carter JD, Frampton CMA, McKenzie JM, Latner JD, et al. Psychotherapy for transdiagnostic binge eating: A randomized controlled trial of cognitive-behavioural therapy, appetite-focused cognitive-behavioural therapy, and schema therapy. Psychiatry Res. 2016;240:412-20.

36. Mitchell JE, Crosby RD, Wonderlich SA, Crow S, Lancaster K, Simonich H, et al. A randomized trial comparing the efficacy of cognitive-behavioral therapy for bulimia nervosa delivered via telemedicine versus face-to-face. Behav Res Ther. 2008;46(5):581-92.

37. Munsch S, Biedert E, Meyer A, Michael T, Schlup B, Tuch A, et al. A randomized comparison of cognitive behavioral therapy and behavioral weight loss treatment for overweight individuals with binge eating disorder. Int J Eat Disord. 2007;40(2):102-13.

38. Olmsted MP. Bulimia nervosa: Minimal treatment and rapid response [Ph.D.]. Ann Arbor: York University (Canada); 1989.

39. Pellizzer ML, Waller G, Wade TD. Ten‐session cognitive behaviour therapy for eating disorders: Outcomes from a pragmatic pilot study of Australian non‐underweight clients. Clinical Psychologist. 2018;23(2):124-32.

40. Pellizzer ML, Waller G, Wade TD. A pragmatic effectiveness study of 10-session cognitive behavioural therapy (CBT-T) for eating disorders: Targeting barriers to treatment provision. Eur Eat Disord Rev. 2019;27(5):557-70.

41. Peterson CB, Mitchell JE, Crow SJ, Crosby RD, Wonderlich SA. The efficacy of self-help group treatment and therapist-led group treatment for binge eating disorder. Am J Psychiatry. 2009;166(12):1347-54.

42. Poulsen S, Lunn S, Daniel SI, Folke S, Mathiesen BB, Katznelson H, et al. A randomized controlled trial of psychoanalytic psychotherapy or cognitive-behavioral therapy for bulimia nervosa. Am J Psychiatry. 2014;171(1):109-16.

43. Quilty LC, Allen TA, Davis C, Knyahnytska Y, Kaplan AS. A randomized comparison of long acting methylphenidate and cognitive behavioral therapy in the treatment of binge eating disorder. Psychiatry Res. 2019;273:467-74.

44. Raykos BC, McEvoy PM, Erceg-Hurn D, Byrne SM, Fursland A, Nathan P. Therapeutic alliance in Enhanced Cognitive Behavioural Therapy for bulimia nervosa: probably necessary but definitely insufficient. Behav Res Ther. 2014;57:65-71.

45. Raykos BC, Watson HJ, Fursland A, Byrne SM, Nathan P. Prognostic value of rapid response to enhanced cognitive behavioral therapy in a routine clinic sample of eating disorder outpatients. Int J Eat Disord. 2013;46(8):764-70.

46. Ricca V, Castellini G, Lo Sauro C, Mannucci E, Ravaldi C, Rotella F, et al. Cognitive-behavioral therapy for threshold and subthreshold anorexia nervosa: a three-year follow-up study. Psychother Psychosom. 2010;79(4):238-48.

47. Riesco N, Aguera Z, Granero R, Jimenez-Murcia S, Menchon JM, Fernandez-Aranda F. Other Specified Feeding or Eating Disorders (OSFED): Clinical heterogeneity and cognitive-behavioral therapy outcome. Eur Psychiatry. 2018;54:109-16.

48. Rigaud DJ, Brayer V, Roblot A, Brindisi MC, Verges B. Efficacy of tube feeding in binge-eating/vomiting patients: a 2-month randomized trial with 1-year follow-up. JPEN J Parenter Enteral Nutr. 2011;35(3):356-64.

49. Rose C, Waller G. Cognitive-behavioral therapy for eating disorders in primary care settings: Does it work, and does a greater dose make it more effective? Int J Eat Disord. 2017;50(12):1350-5.

50. Schlup B, Meyer AH, Munsch S. A non-randomized direct comparison of cognitive-behavioral short- and long-term treatment for binge eating disorder. Obes Facts. 2010;3(4):261-6.

51. Schlup B, Munsch S, Meyer AH, Margraf J, Wilhelm FH. The efficacy of a short version of a cognitive-behavioral treatment followed by booster sessions for binge eating disorder. Behav Res Ther. 2009;47(7):628-35.

52. Tasca GA, Ritchie K, Demidenko N, Balfour L, Krysanski V, Weekes K, et al. Matching women with binge eating disorder to group treatment based on attachment anxiety: outcomes and moderating effects. Psychother Res. 2013;23(3):301-14.

53. Tomba E, Tecuta L, Schumann R, Ballardini D. Does psychological well-being change following treatment? An exploratory study on outpatients with eating disorders. Compr Psychiatry. 2017;74:61-9.

54. Turner H, Bryant-Waugh R, Marshall E. The impact of early symptom change and therapeutic alliance on treatment outcome in cognitive-behavioural therapy for eating disorders. Behav Res Ther. 2015;73:165-9.

55. Valbak K. Good outcome for bulimic patients in long-term group analysis: A single-group study. European Eating Disorders Review. 2001;9(1):19-32.

56. Wade S, Byrne S, Allen K. Enhanced cognitive behavioral therapy for eating disorders adapted for a group setting. Int J Eat Disord. 2017;50(8):863-72.

57. Waller G, Gray E, Hinrichsen H, Mountford V, Lawson R, Patient E. Cognitive-behavioral therapy for bulimia nervosa and atypical bulimic nervosa: effectiveness in clinical settings. Int J Eat Disord. 2014;47(1):13-7.

58. Waller G, Tatham M, Turner H, Mountford VA, Bennetts A, Bramwell K, et al. A 10-session cognitive-behavioral therapy (CBT-T) for eating disorders: Outcomes from a case series of nonunderweight adult patients. Int J Eat Disord. 2018;51(3):262-9.

59. Wonderlich SA, Peterson CB, Crosby RD, Smith TL, Klein MH, Mitchell JE, et al. A randomized controlled comparison of integrative cognitive-affective therapy (ICAT) and enhanced cognitive-behavioral therapy (CBT-E) for bulimia nervosa. Psychol Med. 2014;44(3):543-53.

60. Yu J, Stewart Agras W, Halmi KA, Crow S, Mitchell J, Bryson SW. A 1-year follow-up of a multi-center treatment trial of adults with anorexia nervosa. Eat Weight Disord. 2011;16(3):e177-81.

61. Zerwas SC, Watson HJ, Hofmeier SM, Levine MD, Hamer RM, Crosby RD, et al. CBT4BN: A Randomized Controlled Trial of Online Chat and Face-to-Face Group Therapy for Bulimia Nervosa. Psychother Psychosom. 2017;86(1):47-53.

62. Zipfel S, Wild B, Gross G, Friederich HC, Teufel M, Schellberg D, et al. Focal psychodynamic therapy, cognitive behaviour therapy, and optimised treatment as usual in outpatients with anorexia nervosa (ANTOP study): randomised controlled trial. Lancet. 2014;383(9912):127-37.

**Appendix B: Search strategy**

For the construct “eating disorders”, the terms “eating disorder*”, “anorexia nervosa”, “bulimia” and “binge eating disorder*” were used.

For the construct “cognitive behavior therapy”, the terms “cognitive behavior psychotherapy”, “cognitive behaviour psychotherapy”, “cognitive behaviour therapy”, “cognitive behavior therapy”, “cognitive behavior treatment”, “cognitive behaviour treatment”, “cognitive behavior approach”, “cognitive behaviour approach”, “CBT-E”, “CBT-ED”, “CBT-AN”, “CBT-BN” and “CBT-T” were used.

For the construct “psychodynamic-interpersonal therapy”, the terms “psychodynamic psychotherapy”, “psychodynamic therapy”, “psychodynamic treatment”, “psychodynamic approach”, “mentalization based psychotherapy”, “mentalization based therapy”, “mentalization based treatment” “mentalization based approach”, “interpersonal psychotherapy”, “interpersonal therapy”, “interpersonal treatment”, “interpersonal approach”, “expressive psychotherapy”, “expressive therapy”, “expressive treatment”, “expressive approach”, “supportive psychotherapy”, “supportive therapy”, “supportive treatment”, “supportive approach”, “analytic psychotherapy”, “analytic therapy”, “analytic treatment”, “analytic approach”, “psychoanalytic psychotherapy”, “psychoanalytic therapy”, “psychoanalytic treatment”, “psychoanalytic approach” and “psychoanalysis” were used.

**Appendix C: Funnel plot**

**Figure C1**

*Funnel Plot for Logit Event Rate for Remission by Standard Error of Measurement*

**Appendix D: Publication bias assessment**

Visual inspection of the funnel plot for Logit Event rates for remission (Figure C1) was performed. The studies were symmetrically distributed around the weighted mean effect size, not indicative of small study bias (41). Egger's regression test was not significant (*t(79) =* 1.36, *p* = .178)*,* indicating that sample size did not predict effect size. However, the funnel plot indicated that high-precision studies varied significantly in effect size around the weighted mean. The Duvall and Tweedie`s trim and fill method for imputing studies under the random effects model suggested that no studies were missing for CBT samples. For PIT samples it was suggested that two studies were missing with a higher effect size than the mean. Imputing these studies increased the effect from .216 to .244, 95% CI [.175, .328].

**Appendix E: Primary study characteristics sorted by diagnostic subgroup**

| **Table E1.**  *Study Characteristics for AN Samples (k = 20)* | | | | | | | | |
| --- | --- | --- | --- | --- | --- | --- | --- | --- |
| Study name | Treatment condition | ITT (*n*) | Completers (*n*) | Specific | General | Remission (*n*) | Mean age | Design |
| Ball 2004 | CBT | 13 |  | EDI-BD; EDE-global | BDI; STAI | 7 | 18.5 | RCT |
| Castellini 2015 | CBT | 32 |  |  |  | 21 |  | nRCT |
| Dalle Grave 2013 | CBT | 46 |  | EDE-Q-global | SCL-90 | 13 | 15.5 | nRCT |
| Dalle Grave 2019 | CBT | 49 |  | EDE-Q-global | BSI | 14 | 15.5 | nRCT |
| Dare 2001 | PIT | 21 |  |  |  | 3 | 26.7 | RCT |
| Fairburn 2013 | CBT | 99 |  | EDE-Q-global | GSI | 34 | 24.0 | nRCT |
| Frostad 2018a | CBT | 17 |  |  |  | 5 |  | nRCT |
| Frostad 2018b | CBT | 44 |  |  |  | 17 | 23.3 | nRCT |
| Gowers 1994 | PIT | 20 |  |  |  | 4 | 21.2 | RCT |
| Hay 2018 | CBT | 78 |  | EDE-global | Kessler-10 | 22 | 28.6 | RCT |
| Jenkins | CBT | 63 |  | EDE-global |  | 9 | 23.6 | nRCT |
| La Mela 2013 | CBT | 18 |  |  |  | 6 |  | nRCT |
| Lelli 2019 | CBT | 34 |  |  |  | 15 |  | nRCT |
| Lock 2013 | CBT | 23 |  | EDE-global |  | 11 | 14.7 | RCT |
| Raykos 2013 | CBT | 17 |  |  |  | 7 |  | nRCT |
| Ricca 2010a | CBT (sample 1) | 53 |  |  |  | 19 | 14.7 | RCT |
|  | CBT (sample 2) | 50 |  |  |  | 12 | 23.0 | RCT |
| Sauro 2013 | CBT | 134 |  |  |  | 73 | 27.2 | nRCT |
| Zipfel 2014 | PIT | 80 |  |  |  | 28 | 28.0 | RCT |
|  | CBT | 80 |  |  |  | 15 | 27.4 | RCT |
| *Note.* CBT = cognitive behavior therapy; PIT = psychodynamic-interpersonal therapy; ITT = intention-to-treat sample size; RCT = randomized controlled trial design; nRCT = non-randomized/controlled trial design; EDI = eating disorder inventory; BD = *body dissatisfaction* subscale in EDI; DT = *drive for thinness* subscale in EDI; EDE = eating disorder examination; EDE-Q = EDE questionnaire; BDI = Beck’s depression inventory; SCL-90 = symptom checklist including 90 items; STAI = Stait Trait Anxiety Inventory; Kessler-10 = Kessler psychological disstress scale including 10 items; GSI = *global severity index* for SCL-90. | | | | | | | | |

| **Table E2.**  *Study Characteristics for BED Samples (k = 17)* | | | | | | | | |
| --- | --- | --- | --- | --- | --- | --- | --- | --- |
| Study name | Treatment condition | ITT (*n*) | Completers (*n*) | Specific | General | Remission (*n*) | Mean age | Design |
| Aguera 2013 | CBT | 87 |  |  |  | 41 | 34.1 | nRCT |
| Dingemans 2007 | CBT | 30 |  | EDE-global | BDI | 19 | 38.8 | RCT |
| Fischer 2014 | CBT | 41 |  | EDE-Q |  | 21 | 45.6 | RCT |
| Grilo 2011 | CBT | 45 | 37 | EDE-global | BDI | 23 | 44.8 | RCT |
| Hilbert 2004 | CBT (sample 1) | 14 | 12 | EDE-Q-w; EDE-Q-s;  EDE-Q-e; EDE-Q-r | BDI | 6 | 42.1 | RCT |
|  | CBT (sample 2) | 14 | 12 | EDE-Q-w; EDE-Q-s;  EDE-Q-e; EDE-Q-r | BDI | 8 | 38.6 | RCT |
| Hilbert 2020 | CBT | 37 |  | EDE-global | BDI | 19 | 15.3 | RCT |
| McIntosh 2016 | CBT | 36 | 28 | EDE-global | SCL-90-d | 19 |  | RCT |
| Munsch 2007 | CBT | 44 | 16 | EDE-Q-w; EDE-Q-s;  EDE-Q-e; EDE-Q-r | BDI; BAI | 23 | 44.4 | RCT |
| Olmsted 1989 | CBT | 30 |  |  |  | 9 | 23.7 | RCT |
| Peterson 2009 | CBT | 60 |  | EDE-Q-global | IDS-SR | 31 | 47.1 | RCT |
| Quilty 2019 | CBT | 27 |  | EDE-global |  | 16 | 32.8 | RCT |
| Schlup 2009 | CBT | 18 |  |  |  | 7 | 47.1 | RCT |
| Schlup 2010 | CBT (sample 1) | 40 |  |  |  | 15 | 44.6 | nRCT |
|  | CBT (sample 2) | 36 |  |  |  | 20 | 44.4 | nRCT |
| Tasca 2013 | PIT (sample 1) | 52 | 31 |  | BDI | 13 | 46.2 | nRCT |
|  | PIT (sample 2) | 50 | 24 |  | BDI | 15 | 24.2 | nRCT |
| *Note.* CBT = cognitive behavior therapy; PIT = psychodynamic-interpersonal therapy; ITT = intention-to-treat sample size; RCT = randomized controlled trial design; nRCT = non-randomized/controlled trial design; EDI = eating disorder inventory; EDE = eating disorder examination; EDE-Q = EDE questionnaire; EDE-Q-w = *weight concern* subscale in EDE-Q; EDE-Q-s = *shape concern* subscale in EDE-Q; EDE-Q-e = *eating concern* subscale in EDE-Q; EDE-Q-r = *restraint subscale* in EDE-Q; EDE-Q-ows = *overevaluation of shape and weight scale* in EDE-Q; BDI = Beck’s depression inventory; SCL-90-d = symptom checklist including 90 items *depression subscale*; STAI = Stait Trait Anxiety Inventory; GHQ-a = General health questionnaire *anxiety subscale*; GHQ-d = General health questionnaire *depression subscale*; HAAD = Hospital anxiety and depression scale *anxiety subscale*; HADD **=** Hospital anxiety and depression scale *depression subscale***;** BAI = Beck’s anxiety inventory; IDS-SR = Inventory of Depressive Symptomatology Self Report; CES-D = Center for Epidemiological Studies depression scale. | | | | | | | | |

| **Table E3.**  *Study Characteristics for Mixed Samples (k = 20)* | | | | | | | | |
| --- | --- | --- | --- | --- | --- | --- | --- | --- |
| Study name | Treatment condition | ITT (*n*) | Completers (*n*) | Specific | General | Remission (*n*) | Mean age | Design |
| Allen 2012 | CBT | 43 |  | EDE-global |  | 11 | 26.4 | nRCT |
| Arcelus 2009 | PIT | 59 |  | EDE-global | BDI | 9 | 28.1 | nRCT |
| Bandini 2006 | CBT | 67 |  |  |  | 43 | 20.9 | nRCT |
| Byrne 2011 | CBT | 125 |  | EDE-global | DASS-A; DASS-D | 40 | 26.0 | nRCT |
| Dalle Grave 2015 | CBT | 68 |  | EDE-Q-global | SCL-90 | 25 | 16.5 | nRCT |
| McIntosh 2016 | CBT | 38 | 30 | EDE-global | SCL-90-dep | 16 | 34.4 | RCT |
| Mitchell 2008 | CBT | 66 | 25 | EDE-Q-w; EDE-Q-s;  EDE-Q-e; EDE-Q-r | HAM-D | 13 | 29.6 | RCT |
| Pellizzer 2018 | CBT | 26 |  | EDE-global | DASS-21 | 9 | 28.7 | nRCT |
| Pellizzer 2019 | CBT | 52 |  | EDE-Q-global | DASS | 25 | 26.4 | nRCT |
| Raykos 2013 | CBT | 38 |  |  |  | 23 |  | nRCT |
| Riesco 2018 | CBT (sample 1) | 82 |  |  |  | 8 | 25.2 | nRCT |
|  | CBT (sample 2) | 57 |  |  |  | 10 | 27.4 | nRCT |
|  | CBT (sample 3) | 37 |  |  |  | 8 | 27.4 | nRCT |
| Rigaud 2011 | CBT | 51 |  |  |  | 13 | 28.0 | RCT |
| Rose 2017 | CBT | 47 |  | EDE-Q-global | PHQ-9 | 11 | 27.1 | nRCT |
| Tomba 2017 | CBT | 195 | 185 | EAT |  | 51 | 28.9 | nRCT |
| Turner 2015b | CBT | 179 |  | EDE-Q | HADS-A; HADS-D | 34 | 27.6 | nRCT |
| Wade 2017 | CBT | 39 |  | EDE-global |  | 4 | 23.9 | nRCT |
| Waller 2014 | CBT | 78 |  |  | BDI | 39 | 27.8 | nRCT |
| Waller 2018 | CBT | 93 |  | EDE-Q | BDI | 39 | 27.4 | nRCT |
| *Note.* CBT = cognitive behavior therapy; PIT = psychodynamic-interpersonal therapy; ITT = intention-to-treat sample size; RCT = randomized controlled trial design; nRCT = non-randomized/controlled trial design; EDI = eating disorder inventory; BD = *body dissatisfaction* subscale in EDI; DT = *drive for thinness* subscale in EDI; EDE = eating disorder examination; EDE-Q = EDE questionnaire; EDE-Q-w = *weight concern* subscale in EDE-Q; EDE-Q-s = *shape concern* subscale in EDE-Q; EDE-Q-e = *eating concern* subscale in EDE-Q; EDE-Q-r = *restraint subscale* in EDE-Q; BDI = Beck’s depression inventory; SCL-90 = Symptom checklist including 90 items; SCL-90-d = SCL-90 *depression subscale;* HADS-A = Hospital anxiety and depression scale *anxiety subscale*; HADS-D **=** Hospital anxiety and depression scale *depression subscale***;** GAD-A = General anxiety disorder assessment; PHQ-9 = Patient health questionnaire *major depression module*; DASS-A = Depression anxiety stress scale *anxiety subscale*; DASS-D = Depression anxiety stress scale *depression subscale*; HAM-A = Hamilton *anxiety* rating scale; HAM-D = Hamilton *depression* rating scale; BSI = Brief symptom inventory; CC-total = Cognitive checklist for *anxiety* and *depression*. | | | | | | | | |

| **Table E4.**  *Study Characteristics for BN Samples (k = 19).* | | | | | | | | |
| --- | --- | --- | --- | --- | --- | --- | --- | --- |
| Study name | Treatment condition | ITT (*n*) | Completers (*n*) | Specific | General | Remission (*n*) | Mean age | Design |
| Agras 2000a | CBT | 194 |  |  |  | 58 | 28.4 | RCT |
| Agras 2000b | PIT | 110 | 64 | EDE-global | SCL-90 | 17 | 27.9 | RCT |
|  | CBT | 110 | 65 | EDE-global | SCL-90 | 29 | 28.3 | RCT |
| Aguera 2013 | CBT (sample 1) | 327 |  |  |  | 89 | 26.2 | nRCT |
|  | CBT (sample 2) | 40 |  |  |  | 12 | 27.2 | nRCT |
| Bailer 2004 | CBT | 41 | 26 | EDI-BD; EDI-DT | BDI | 6 | 24.2 | RCT |
| Chen 2003 | CBT (sample 1) | 30 |  | EDE-global | BDI; STAI-S; STAI-T | 4 | 25.8 | RCT |
|  | CBT (sample 2) | 30 |  | EDE-global | BDI; STAI-S; STAI-T | 3 | 25.8 | RCT |
| Cooper 1995 | CBT | 13 | 12 | EDE-r; EDE-s; EDE-w | BDI; STAI-S; STAI-T | 6 | 23.8 | RCT |
| Garner 1993 | CBT | 25 |  | EDE-r; EDE-s; EDE-w | BDI | 9 | 23.7 | RCT |
|  | PIT | 25 |  | EDE-r; EDE-s; EDE-w | BDI | 3 | 24.6 | RCT |
| Goldbloom 1997 | CBT | 24 | 13 | EDE-Q-s; EDE-Q-w | BDI | 6 | 25.8 | RCT |
| Le Grange 2015 | CBT | 58 | 40 |  | BDI | 19 | 15.7 | RCT |
| Poulsen 2014 | CBT | 36 |  |  |  | 15 | 25.8 | RCT |
|  | PIT | 34 |  |  |  | 2 | 25.8 | RCT |
| Raykos 2013 | CBT | 50 |  |  |  | 17 |  | nRCT |
| Valbak 2001 | PIT | 19 | 10 |  | SCL-90 | 9 |  | nRCT |
| Wonderlich 2014 | CBT | 40 |  | EDE-global | BDI; STAI | 9 | 28.8 | RCT |
| Zerwas 2016 | CBT | 90 |  |  |  | 27 | 27.5 | RCT |
| *Note.* CBT = cognitive behavior therapy; PIT = psychodynamic-interpersonal therapy; ITT = intention-to-treat sample size; RCT = randomized controlled trial design; nRCT = non-randomized/controlled trial design; EDI = eating disorder inventory; BD = *body dissatisfaction* subscale in EDI; DT = *drive for thinness* subscale in EDI; EDE = eating disorder examination; EDE-Q = EDE questionnaire; EDE-Q-w = *weight concern* subscale in EDE-Q; EDE-Q-s = *shape concern* subscale in EDE-Q; EDE-Q-r = *restraint subscale* in EDE-Q; EAT-26 = Eating attitude test 26 items; BDI = Beck’s depression inventory; SCL-90 = Symptom checklist including 90 items; SCL-90-a = SCL-90 *anxiety subscale*; SCL-90-d = SCL-90 *depression subscale*; STAI = Stait Trait Anxiety Inventory; STAI-t = STAI *trait* subscale; STAI-s = STAI *state* subscale; MADRS = Montgomery Aasberg Depression Rating Scale; IDA-a = Irritability Depression and Axiety Scale *anxiety* subscale; IDA-d = Irritability Depression and Anxiety Scale *depression* subscale. | | | | | | | | |

**Appendix F: Primary study quality assessment**

| **Table F1.**  *Quality assessment for AN Studies (k = 17) by treatment condition and risk of bias domain* | | | | | | | | | | |
| --- | --- | --- | --- | --- | --- | --- | --- | --- | --- | --- |
| Study name | Treatment condition | Selection bias | Design | Confounders | Blinding | Data collection | Drop-out | Integrity | Analyses | Global rating |
| Ball 2004 | CBT | * | ** | *** | ** | *** | ** | *** | *** | ** |
| Castellini 2015 | CBT | * | ** | * | ** | *** | * | ** | * | * |
| Dalle Grave 2013 | CBT | ** | ** | ** | ** | *** | ** | *** | *** | *** |
| Dalle Grave 2019 | CBT | ** | ** | ** | ** | *** | ** | *** | *** | *** |
| Dare 2001 | PIT | *** | *** | *** | ** | *** | ** | *** | *** | *** |
| Fairburn 2013 | CBT | ** | ** | ** | ** | *** | ** | *** | *** | *** |
| Frostad 2018a | CBT | ** | ** | ** | ** | *** | * | *** | *** | ** |
| Gowers 1994 | PIT | * | *** | ** | ** | *** | *** | *** | *** | ** |
| Hay 2018 | CBT | * | *** | *** | *** | *** | *** | *** | *** | ** |
| Jenkins 2019 | CBT | ** | ** | ** | ** | *** | ** | *** | *** | *** |
| La Mela 2013 | CBT | * | ** | ** | ** | *** | ** | *** | *** | ** |
| Lelli 2019 | CBT | * | ** | ** | ** | *** | * | *** | *** | * |
| Lock 2013 | CBT | ** | *** | *** | *** | *** | ** | *** | *** | ** |
| Raykos 2013 | CBT | * | ** | ** | ** | *** | ** | *** | *** | ** |
| Ricca 2010a | CBT | ** | ** | ** | ** | *** | ** | *** | *** | *** |
| Lo Sauro 2013 | CBT | *** | ** | ** | ** | *** | *** | *** | *** | *** |
| Zipfel 2014 | CBT/PIT | ** | *** | ** | ** | *** | ** | *** | *** | *** |
| *Note:* CBT= Cognitive behavior therapy, PIT= Psychodynamic interpersonal therapy, Quality ratings: ***= Strong **= Moderate * Weak, AN= Anorexia Nervosa | | | | | | | | | | |

| **Table F2.**  *Quality assessment for BED Studies (k = 14) by treatment condition and risk of bias domain* | | | | | | | | | | |
| --- | --- | --- | --- | --- | --- | --- | --- | --- | --- | --- |
| Study name | Treatment condition | Selection bias | Design | Confounders | Blinding | Data collection | Drop-out | Integrity | Analyses | Global rating |
| Aguera 2013 | CBT | ** | ** | ** | * | *** | ** | *** | *** | ** |
| Dingemans 2007 | CBT | ** | *** | *** | *** | *** | *** | *** | *** | *** |
| Fischer 2014 | CBT | ** | *** | * | ** | *** | *** | *** | *** | ** |
| Grilo 2011 | CBT | * | *** | *** | ** | *** | *** | *** | *** | ** |
| Hilbert 2020 | CBT | ** | *** | * | ** | *** | ** | *** | *** | ** |
| Hilbert 2004 | CBT | * | *** | ** | ** | *** | *** | *** | *** | ** |
| McIntosh 2016 | CBT | ** | *** | *** | ** | *** | ** | *** | *** | *** |
| Munsch 2007 | CBT | * | *** | *** | *** | *** | ** | *** | *** | ** |
| Olmsted 1989 | CBT | ** | ** | ** | ** | *** | ** | *** | *** | *** |
| Peterson 2009 | CBT | * | *** | *** | *** | *** | ** | *** | *** | ** |
| Quilty 2019 | CBT | * | *** | *** | *** | *** | ** | *** | *** | ** |
| Schlup 2010 | CBT | ** | ** | *** | ** | *** | ** | *** | *** | *** |
| Schlup 2009 | CBT | * | ** | ** | ** | *** | ** | *** | *** | ** |
| Tasca 2013 | PIT | ** | ** | ** | ** | *** | *** | *** | *** | *** |
| *Note:* CBT= Cognitive behavior therapy, PIT= Psychodynamic interpersonal therapy, Quality ratings: ***= Strong **= Moderate * Weak, BED= Binge eating disorder | | | | | | | | | | |

| **Table F3.**  *Quality assessment for Mixed Studies (k = 18) by treatment condition and risk of bias domain* | | | | | | | | | | |
| --- | --- | --- | --- | --- | --- | --- | --- | --- | --- | --- |
| Study name | Treatment condition | Selection bias | Design | Confounders | Blinding | Data collection | Drop-out | Integrity | Analyses | Overall |
| Allen 2012 | CBT | * | ** | *** | ** | *** | * | *** | *** | * |
| Arcelus 2009 | PIT | * | ** | ** | ** | *** | ** | *** | *** | ** |
| Bandini 2006 | CBT | ** | ** | ** | ** | *** | ** | *** | *** | ** |
| Byrne 2011 | CBT | ** | ** | ** | ** | *** | * | *** | *** | ** |
| Dalle Grave 2015 | CBT | *** | ** | ** | ** | *** | ** | *** | *** | *** |
| McIntosh 2016b | CBT | ** | *** | *** | ** | *** | ** | *** | *** | *** |
| Mitchell 2008 | CBT | *** | *** | *** | *** | *** | * | *** | *** | ** |
| Pellizzer 2018 | CBT | *** | ** | ** | ** | *** | ** | *** | *** | *** |
| Pellizzer 2019 | CBT | *** | ** | ** | ** | *** | ** | *** | *** | *** |
| Raykos 2013b | CBT | * | ** | ** | ** | *** | ** | *** | *** | ** |
| Riesco 2018 | CBT | * | ** | ** | ** | *** | * | *** | *** | * |
| Rigaud 2011 | CBT | * | *** | *** | ** | *** | *** | *** | *** | ** |
| Rose 2017 | CBT | * | ** | ** | ** | *** | ** | *** | *** | ** |
| Tomba 2017 | CBT | *** | ** | ** | ** | *** | ** | *** | *** | *** |
| Turner 2015b | CBT | *** | ** | ** | ** | *** | * | *** | *** | ** |
| Wade 2017 | CBT | *** | ** | ** | ** | *** | ** | *** | *** | ** |
| Waller 2014 | CBT | ** | ** | ** | ** | *** | *** | *** | *** | *** |
| Waller 2018 | CBT | ** | ** | ** | ** | *** | ** | *** | *** | ** |
| *Note:* CBT= Cognitive behavior therapy, PIT= Psychodynamic interpersonal therapy, Quality ratings: ***= Strong **= Moderate * Weak, | | | | | | | | | | |

| **Table F4.**  *Quality assessment for BN Studies (k = 15) by treatment condition and risk of bias domain* | | | | | | | | | | |
| --- | --- | --- | --- | --- | --- | --- | --- | --- | --- | --- |
| Study name | Treatment condition | Selection bias | Design | Confounders | Blinding | Data collection | Drop-out | Integrity | Analyses | Overall |
| Agras 2000a | CBT | ** | ** | ** | * | *** | *** | *** | *** | ** |
| Agras 2000b | CBT/PIT | ** | ** | * | *** | *** | ** | *** | *** | ** |
| Aguera 2012 | CBT | * | * | * | * | *** | * | *** | *** | * |
| Aguera 2013b | CBT | ** | ** | ** | * | *** | *** | *** | *** | ** |
| Bailer 2004 | CBT | *** | *** | ** | * | *** | ** | *** | *** | ** |
| Chen 2003 | CBT | ** | *** | *** | ** | *** | ** | *** | *** | *** |
| Cooper 1995 | CBT | * | ** | * | ** | *** | ** | *** | *** | * |
| Garner 1993 | CBT/ PIT | * | *** | ** | *** | *** | *** | *** | *** | ** |
| Goldbloom 1997 | CBT | * | *** | ** | ** | *** | * | *** | *** | * |
| Le Grange 2015 | CBT | ** | *** | ** | ** | *** | ** | *** | *** | *** |
| Poulsen 2014 | CBT/PIT | * | *** | *** | ** | *** | ** | *** | *** | ** |
| Raykos 2013 | CBT | * | ** | ** | ** | *** | *** | *** | *** | ** |
| Valbak 2001 | PIT | * | * | ** | ** | *** | * | ** | *** | * |
| Wonderlich 2014 | CBT | *** | *** | *** | *** | *** | ** | *** | *** | *** |
| Zerwas 2016 | CBT | *** | *** | *** | ** | *** | ** | *** | *** | *** |
| *Note:* CBT= Cognitive behavior therapy, PIT= Psychodynamic interpersonal therapy, Quality ratings: ***= Strong **= Moderate * Weak, BN= Bulimia nervosa | | | | | | | | | | |
